# Proceedings of Veterinary Societies

**Published:** 1889-07

**Authors:** 


### PROCEEDINGS OF VETERINARY SOCIETIES.

THE NEW JERSEY STATE VETERINARY SOCIETY held a fairly well attended meeting at the Continental Hotel, Newark, N. J., at 2.30 P. M., on Thursday, May 16th, 1889.

It was decidéd to hold but two meetings each year, one in August and one in November.

Dr. A. T. Sellers, of Camden, and Dr. E. A. Vreeland, of Jersey City, were elected members. Dr. James McCaffrey, Dr. J. Hopkins, and Dr. M. Dimond, were proposed for membership, and were referred to the Board of Censors.

Dr. J. Nayler read a paper on "Specific Medication versus Polypharmacy," and Dr. W. H. Lowe gave some interesting facts regarding the law which has been passed by the Legislature last March, both received a vote of thanks.

The next meeting will be held in New Brunswick, and it being the annual meeting new officers are to be elected.

CHAS. KUEHNE, *Secretary*.

MONTREAL VETERINARY COLLEGE.—The Twenty-second Session was concluded by the final examination of the students by a Board of Examiners appointed by the Council of Agriculture. The Board was composed of Messrs. J. W. Gadsden, M.R.C.V.S., Philadelphia; Williamson Bryden, V. S., Boston, Mass. ; Arch'd. McCormick, V. S., Ormstown ; J. A. Couture, V.S., Quebec; A. W. Harris, Ottawa, and Dr. James Bell, Montreal. The following students passed successfully the examinations prescribed by the curriculum of the college in the following subjects, viz : Botany, histology, chemistry, physiology, materia medica, anatomy, cattle pathology, and practice and theory of veterinary medicine and surgery, and were considered by the Board fully qualified to practice the art and science of veterinary medicine and surgery, viz : Messrs. R. D. Austin, R. Darling, G. P. Dillon, J. G. Goddard, J. G. Harris, J. McCurdy, H. McWhennie, R. C. Mylne, J. M. Parker, Wm. M. Simpson, T. W. Skaife and E. I. Willand.

The closing exercises took place in the college lecture hall. Col. Rhodes, Minister of Agriculture, occupied the chair, and on his right sat Sir Wm. Dawson, Chancellor of McGill University. Among those present were Messrs. S. M. Blackwood, member of Council of Agriculture; Prof. Wesley Mills, Prof. Penhallow, Dr. McEachren, Dr. Baker, J. X. Perrault, and a number of others interested in the work of the college. Col. Rhodes proceeded, amid continued applause, to award the prizes to the successful competitors. The following is a list of the prizes awarded :

Silver medal, the gift of the Council of Agriculture, for the highest nnmber of marks in all the subjects for the three years. Won by Mr. Robert Darling.

PRACTICE OF MEDICINE AND SURGERY.—First prize, J. G. Harris; 2d prize, E. A. Wieland.

CATTLE PATHOLOGY.—First prize, E. A. Wieland; 2d prize, H. McWhinnie.

ANATOMY.—First prize, Wm. M. Simpson; 2d prize, H. McWhinnie.

Special prize given by Prof. Wesley Mills, for his efforts in connection with the study of comparative psychology, to J. M. Parker.

Prize awarded by Prof. J. A. Couture, for best oral examination in the practice of medicine and anatomy, won by Henry McWhinnie.

JUNIORS.—Physiology—First, R. N. Walsh; 2d, J. F. Scott.

Chemistry—First, J. F. Scott; 2d, M. Hayman.

Materia Medica—First, A. M. York and J. F. Scott, equal; 2d, R. N. Walsh.

Anatomy—First, A. M. York.

The prizes having been awarded, Sir Wm. Dawson was called on, and at the commencement of his address, referred very touchingly to the death of Dr. R. P. Howard, Dean of the Medical Faculty of McGill. He spoke of the deceased as a man of excellent qualities, and felt deeply grieved at his death. The veteran Chancellor of McGill paid a high compliment to the Montreal Veterinary College, and said it was a valued and important institution, and deserved all credit in the work which is of a highly practical kind. He was glad that McGill was able to do something towards carrying on this important work. He believed that McGill would welcome closer connection with the Veterinary College, recognizing the growing importance of veterinary science. Personally, he was much in sympathy with the school, whose studies occupied a very high place in practical science.

Col. Rhodes, addressed the assemblage, and in the course of his remarks said that the education of men in agricultural pursuits is a very important study. He said he owed the position he now holds to his knowledge of all the details of agriculture. He stated that the Provincial Government wants to improve the position of the veterinary profession, and expressed a desire to have the Veterinary College more closely allied with the Agricultural Department of the Government, which is a most natural position for it to hold, and the outcome would be very beneficial. He offered to the College the services of the Provincial lecturers on agricultural branches, should their services at any time be needed by the College. The gallant Colonel promised to give his hearty support to the institution, both in his official capacity and as a private citizen.

Mr. S. M. Blackwood, a member of the Council of Agriculture, and one of the examiners, spoke highly of the graduating class. He commented on the fact that too many of our veterinary surgeons went abroad when their services were much required at home. He said some effort should be made to keep the graduates at home.

Mr. J. W. Gadsden congratulated the men on the profession they had chosen and on the school they had selected wherein to pursue their studies.

As one of the examiners, he spoke very highly of the manner in which the graduating class had acquitted themselves in their examinations. He denounced the manner in which some so-called American colleges granted Diplomas to men who had been able only to get a mere smattering of the knowledge required by a Veterinary Surgeon, and characterized it as very unfair and thought some change should be effected so as to guard the public and the profession as well.

Mr. Williamson Bryden, eulogized the Montreal Veterinary College as having attained an eminent position among colleges of its kind in the country.

Mr. F. W. Skaife, one of the graduates made the Valedictory address, which was a good one, the subject being treated as viewed by the men who are about to go forth to enter upon the duties of their profession. The address treated the veterinary question on a commercial basis, and dwelt on some advantages in that line. It also treated the veterinary profession in its relation to and standing among the sciences. The study of human and animal life could not easily be separated, and as the study of the latter becomes more advanced, the relation of the two becomes more apparent. The address also treated the profession and its relation to society. The valedictorian was greeted with hearty applause from the students benches, as well as from the platform.

Dr. McEachran said the Veterinary College was much indebted to McGill University and the Provincial Government for aid in carrying on the work, and without which they would be sadly off. He said it would be a great honour for the College to be taken under the wing of McGill and made a faculty of that University.

Professors Wesley Mills and Penhallow were called for and both responded by making a few remarks. Professor Mills spoke very highly of a paper on Comparative Psychology by Mr. J. M. Parker, for which he received a prize.

---

ONTARIO VETERINARY COLLEGE.—The closing exercises of this college took place on the 29th day of March. For some three weeks previous to that date the examinations, written and oral, had been going on. The Board of Government Examiners had conducted the oral examinations, and tested very thoroughly the numerous applicants for the diplomas. About 130 gentlemen succeeded in passing. The honor list was also a long one testifying to the faithful work which both students and teachers had performed during the winter.

The proceedings in connection with the closing exercises took place in Richmond Hall. Professor Smith, F. R. C. V. S., Principal of the College, presided. There was a large attendance of students; and many friends of the College were present, among whom may be mentioned Sir Daniel Wilson, President of University College, Hon. Charles Drury, Minister of Agriculture, J. J. Witherow, Esq., President of the Industrial Exhibition Association, Ald. Dodds, Alderman Frankland, the members of the Board of Examiners, the members of the Faculty of the College, and others.

Professor Smith, in opening the proceedings, referred to the successful session that was just closing. There had been a large attendance of students from all parts of the Dominion, as also from nearly every State of the Union, and one from no less distant a country than the Sandwich Islands. About 400 students had been in attendance during the session.

The Prize and Honor list was then read by Dr. Duncan. Sir Daniel Wilson was next called upon. He congratulated Professor Smith on the high state of efficiency to which the Veterinary College had attained, and expressed his earnest desire that it might go on and prosper. It was an institution that Toronto had every reason to be proud of. Hon. C. Drury presented the gold medal of the Agriculture and Arts Association to the winner, Mr. E. A. Sturge, of London, England, and alluded to the progress of Canada from an agricultural point of view; and to the immense amount of money invested in live stock, which amounted in cattle alone to about $100,000,000, which pointed to the fact that Canada was fast becoming one of the most important cattle raising countries in the world.

Mr. J. J. Witherow referred to the fact that a new college building was to be immediately erected. He offered his congratulations on this evidence of the growth and importance of the College.

Alderman Frankland spoke eloquently and in complimentary terms of the College, its President, Faculty, graduates, and students. He set before the students the importance of seizing on and improving every opportunity for improvement and advancement.

Alderman Dodds referred to the value of the Diploma of this College, as shown during a recent trip of his through a large portion of the United States. He found that its possession was a passport to the confidence of the better class of stockmen in all parts of that country.

Among other speakers were Major Lloyd, Dr. Thorburn, and members of the Board of Examiners, who presented the various prizes to the successful students. The prize list is as follows:

SENIORS.

*Pathology.*—Silver medal—J. E. Duncan. Second prize—E. Sturge, H. H. Jenkins (equal). Third prize—F. M. Hopkins, J. D. Nighbert (equal).

*Anatomy.*—Silver medal—E. Sturge. Second prize—H. H. Jenkins. Third prize—J. E. Duncan.

*Entozoa.*—First prize—H. H. Jenkins and E. Richardson (equal).

*Dissected Specimens.*—Gold medal, given by the Toronto Industrial Exhibition Association, awarded to F. J. Gallanough, Thornhill, Ont. Second prize—$30—J. D. G. Warwick. Third prize—$20—D. W. Rose.

*Microscopy.*—First prize—J. Manchester. Second Prize—H. H. Jenkins and E. Sturge (equal). Third prize—P. Thwaites.

*Physiology.*—Silver medal—H. H. Jenkins. Second prize—E. Sturge. Third Prize—A. C. Lloyd.

*Materia Medica.*—First prize—H. H. Jenkins. Second prize—E. Sturge. Third prize—C. W. Purcell.

*Best General Examination.*—Gold medal, given by the Ontario Veterinary Medical Association, awarded to E. Sturge.

JUNIORS.

*Anatomy.*—Silver medal—W. Wooton. Second prize—J. H. Ulrich Third prize—R. E. Cooper, M. H. Davitt, E. Jupp (equal).

*Pathology.*—First prize—O. L. Boor, J. H. Ulrich, P. R. Sidebottom (equal). Second prize—A. J. Terry, L. A. Wright (equal). Third prize—G. R. Teeple.

*Physiology.*—First prize—J. J. Fyle. Second prize—A. L. Boor. Third prize—J. A. Kelly, E. Wilson (equal).

GRADUATES.

Adams, Herbert Turgeant, Clarksville, Howard county, N. W. T.; Alexander, Thomas J., Strathroy, Ontario; Alton, William Wellesley, Appleby, Ont; Alverson, Alfred G., Cherry Valley, Ill., U. S.; Bowman, Robert C., Ilderton, Ont.; Bingham, James Edgar, Tyrone, Ont.; Bullivant, James, Tampa, Florida, U. S.; Bock, Aaron R., New Dundee, Ont.; Barnett, Frank E., West Salem, Wayne county, Ohio; Brown, Leopold Alexander, Dunboyne, Ont.; Baker, Lewis R., Wannakee, Wis., U. S.; Bechtel, Milton T., Waterloo, Ont.; Burgess, Herbert W., Bennington, Vt., U. S.; Boucher, William Woods, South March, Ont.; Butler, W. J., Stirling, Ont.; Brindle, D. C., Chambersburg, Pa. U. S.; Beattie, Francis Scott, Seaforth, Ont.: Blanchard, William Hutchinson, Pocklington, England; Buckham, James, Brampton, Ont.; Blacklinton, Joseph C., Bate, Ohio, U. S.; Campbell, Andrew; Cassels, William G., Paisley, Ont.; Campbell, John R., Milton, Ont.; Campbell, Peter M., Strathroy, Ont.; Church, Joseph Alexander; Callander, J. C., Smith's Falls, Ont.; Craig, William B., Indianapolis, Ind., U. S.; Donaldson, Thomas Alexander; Doan, Berkley Potts, Port Dover, Ont.; Duncan, James Edward, Canandaigua, N. Y., U. S.; Dunn, William H., Riga, N. Y., U. S.; Dorney, Albert H., Allentown, Pa., U. S.; Dewey, David H., North Manlins, N. Y., U. S.; Duncombe, Orlando Hardy, Waterford, Ont.; Detwiler, Charles H., Iron Bridge, Montgomery county, Pa.; Doswell, A., Toronto, Ont.; Diggs, Edward F., Winchester, Ind., U. S.; Eaid, Charles E., Jarvis, Ont.; Eisenhart, Oscar C., Bingen, Penn., U. S.; Fisher, George Edward, Goderich, Ont.; Falconer, Charles, Kendall, N. Y., U. S.; Franks, J. W.; Glendinning, C. G., Belfountain, Ont.; Gilchrist, William P., Fort Edward, N. Y., U. S.; Grieve, John, Seaforth, Ont.; Gordon, D. Baillie, Ottawa, Ont.; Gallanough, Fred. J., Thornhill, Ont.; Greenwood, John, Wellesley, Ont.; Hutton, Frederick G., Welland, Ont.; Hopkins, Frank M., Topeka, Kan., U. S., Hodges, Alfred M., Nanticoke, Ont.; Holbrook, John A., Townshend, Vermont, U. S.; Henry, Elias Wetmore, Fredericton, N. B.; Harrington, John Beverley, Port Arthur, Ont.; Hill, Joseph G., Sennett, N. Y., U. S.; Hamilton, William, St. Mary's, Ont.; Hougendobler, J. J., Rohrerstown, Pa., U. S.; Higbee, William F., Youngstown, N. Y., U. S.; Howard, Samuel Rogers, Circleville, Ohio; Ide, Almon H., East Shelby, N. Y., U. S.; Jenkins, Henry H., N. W. M., Police, North-West Ter.; Jameson. John W., Paris, Ky., U. S.; Johnston, William J., Mine sing, Ont.; King, Thomas, Bluevale, Ont.; Kuhn, John Miller, Mercersburg, Pa., U. S.; Kurtz, Alfred, Neenah, Wis.. U. S.; Leach, Maurice Mackenzie, Paris, Ont.; Lloyd, Arthur C., Detroit, Mich., U. S.; Leslie, Henry Charles, Canton, Ont.; Mossom, Dundas H. D. McQ., London, Eng.; Mullin, D. V., Montreal, Que.; Monserrat, W. T., Honolulu, Sandwich Islands; Marshall, Joseph W., Forest, Ont.; Manchester, John William, Sussex Vale, N. B.; Morrison, William McLeod, Birtle, Man.; Murray, Henry B., Port Albert, Ont.; McIntosh, Archibald J., Toronto, Ont.; McMurtry, D. Henry, South March, Ont.; McGregor, Charles F. Mortimere, Constance, Ont.; McQuate, Theodore C., Canton, Ohio, U. S.; McDonald, John, Petrolea, Ont.; McGahey, Robert P., Kemptville, Ont.; McMicken, William Bell, Chesterfield, Ont.; McBeath, Alonzo E., Bradford, Ont.; McMurtry, W. Randolph, South March, Ont.; McCray, W. E., Oil City, Pa., U. S.; Nighbert, James D., Palmyra, Ill., U. S.; Old, William R. J., Goderich, Ont.; Orr, C. H., Cairo, Mich., U. S.; Pickering, William H., Forest, Ont.; Paul, Bert E., Wayland, Mich., U. S.; Poe, John Julius Evans, Harley Park, Callow, Ireland ; Purcell, Charles Wilson, East Boston, Mass.; Petrie, William, Watertown, N. Y., U. S.; Quantz, Jacob D., Bellantrae, Ont.; Rich, Frank Abiram, Avon, N. Y., U. S.; Rishel, Edward Ira, Vicksburg, Kalamazoo County, Mich.; Rose, D. W., Toronto, Ont.; Robertson, Gilbert James, Beatrice, Neb., U. S.; Rike, Harry W., Dayton, Ohio, U. S.; Spicer, Charles A., Pittsburg, Pa., U. S.; Simons, Frank W., Marengo, Ohio, U. S.; Smith, Charles H., Ansonia, Conn., U. S.; Sturge, Edgar, Guelph, Ont.; Smith, Henry Stephen, Albion, Mich., U. S.; Shevalier, Eugene D., Courtlandt, N. Y., U. S.; Swingley, Jacob G., Oregon, Ill., U. S.; Stevenson, William S., Tyre, N. Y., U. S.; Sutterby, Joseph, Batavia, N. Y., U. S.; Stutzman, Benjamin F., Chappel, Neb., U. S.; Storey, John T., Goodwood, Ont.; Spensley, F. T., Granger, Ohio, U. S.; Saylor, David S., Wellington, Ont. ; Sherrick, Harry R.; Tully, John Walter, Chesley, Ont.; Tanner, Byron L., Mount Forest, Ont.; Tanner, Vassar E., Mount Forest, Ont.; Thwaites, Percy, Toronto, Ont.; Warwick, John D., Wingham, Ont.; Wiley, Horace H., Rochester, Mich., U. S.; Williams, Fred. Erwaat, Burdette, N. Y. U.S.; Wilson, Purvis O., Drumbo, Ont.; Waller, Harry Noel, Prairie Club, Semars, Iowa.; Ward, James R., Alton, Ont.; Wilson, John, Leamington, Ont.

PRIMARY EXAMINATION.

Materia Medica—Bates, Frank ; Carson, David J. ; Keogh, W.

Anatomy—Anderson, Frank H. ; Black, J. F. ; Hassard, Thomas Henry ; Howson, Charles A. ; Johnston, Robert ; Hammond, Wm. D. ; Lees, A. F. ; Vail, Albert E. ; Wilkinson, J. K. ; Edwards, F. H. P.

Messrs. F. H. Anderson and F. H. P. Edwards passed with great credit.

PRESENTATION TO PROF. SMITH.

A pleasant feature in the proceedings was the presentation of a large picture of the graduates in group form to Professor Smith. The picture, which is about six feet square with gilt bronze frame, contained the likeness of 170 graduates. Mr. Montserrat made the presentation, hoping the recipient might long be spared to preside over the institution. Professor Smith suitably replied.

VETERINARY DEPARTMENT UNIVERSITY OF PENNSYLVANIA, held its third annual commencement at the Academy of Music, Philadelphia, on June 5, 1889, The following gentlemen received the degree of V.M.D. :—

Simon D. Brunhall, Minneapolis, Minn.; George Frank Harker, Wrightstown, N. J.; Frank H. Mackie, Fairhill, Md.; Chalkley H. Magill, Philadelphia, Pa.; William H. Mattson, Ward, Pa,; James C. McNeil, Pittsburgh, Pa.; Samuel B. Willard, Yardley, Pa.

---

SOCIETY FOR THE STUDY OF COMPARATIVE PSYCHOLOGY.—At a meeting held at the Montreal Veterinary College, a paper was read entitled "Some thoughts on instinct and reason in man and other animals," by John M. Parker. V.S.

The writer thinks if more interest were taken on the subject, important truths would be learned. *Magister dixit* should not influence people. They should investigate and learn to think for themselves. He gives definitions by best authorities of instinct and reason. Animals he says should not be judged incapable of reasoning because they do many things instinctively; man is often ruled by instinct but reasons nevertheless.

The common opinion that man's superiority over the animals is due to his possessing reasoning powers while the latter possess only instinct is a grave error, says the writer, the truth being that much of the difference is due to education and evolution. He objects to Lubock's statement that "dogs cannot be intelligent unless they know and understand what we know and understand." Animals should be bred for their mental qualities as much as for their physical points. A low bred man is inferior to a well bred brute. He gives many interesting stories to illustrate his point.

He thinks that living creatures possess instinct and reason in an increase ratio, descending the scale they depend on instinct; ascending, their reasoning powers increase.

No animal can be trained unless he can draw conclusions from past experiences and act on them, thereby exercising memory, judgment and *reason.*

